# Supplementary material for: Hypercapnic warm-up and re-warm-up–A novel experimental approach in swimming sprint
Source: PLoS One. 2025 Jan 29;20(1):e0314089. doi: 10.1371/journal.pone.0314089 (PMC11778800; doi:10.1371/journal.pone.0314089)
Supplement: S1 Table — Note: Data presented as mean ± standard deviation. *Significant difference at p < 0.05. PImax I—maximal inspiratory muscle strength at rest, PImax IV—maximal inspiratory muscle strength after the test, PEmax I—maximal expiratory muscle strength at rest, PEmax IV—maximal expiratory muscle strength after the test. WUCON—warm-up in water, WUARDS—warm-up in water with ARDS, RE-WUARDS—warm-up in the water with application of ARDS on land during the transition phase between warm-up and swimming test. (DOCX) [file pone.0314089.s001.docx]

# Supporting information

**Table 1A. Changes in the maximal strength of the inspiratory and expiratory muscles at rest and after the end of the 50 m time trial in the tested warm-up protocols.**

| **Variables** | **Protocol** | **Mean** | **SD** | **ANOVA 95 % confidence interval** | | | **Post-hoc comparison** | | |
| --- | --- | --- | --- | --- | --- | --- | --- | --- | --- |
|  |  |  |  | **F** | ***p*** | **η**^2^ | ***p*** | | |
|  |  |  |  |  |  |  | **1 vs 2** | **1 vs 3** | **2 vs 3** |
| **PImax_I_ (cmH_2_O)** | **WU_CON_** | 140.25 | 26.48 | 1.37 | 0.28 | 0.16 | 1.00 | 0.84 | 1.00 |
|  | **WU_ARDS_** | 140.63 | 22.31 |  |  |  |  |  |  |
|  | **RE-WU_ARDS_** | 142.38 | 28.99 |  |  |  |  |  |  |
| **PImax_IV_ (cmH_2_O)** | **WU_CON_** | 135.75 | 20.39 | 0.30 | 0.60 | 0.04 | 1.00 | 1.00 | 0.59 |
|  | **WU_ARDS_** | 130.50 | 23.91 |  |  |  |  |  |  |
|  | **RE-WU_ARDS_** | 138.38 | 25.76 |  |  |  |  |  |  |
| **PEmax_I_ (cmH_2_O)** | **WU_CON_** | 173.13 | 35.18 | 1.12 | 0.33 | 0.14 | 0.12 | 0.98 | 1.00 |
|  | **WU_ARDS_** | 157.38 | 31.56 |  |  |  |  |  |  |
|  | **RE-WU_ARDS_** | 165.50 | 31.67 |  |  |  |  |  |  |
| **PEmax_IV_ (cmH_2_O)** | **WU_CON_** | 171.25 | 26.62 | 0.68 | 0.44 | 0.09 | 1.00 | 1.00 | 1.00 |
|  | **WU_ARDS_** | 165.88 | 28.69 |  |  |  |  |  |  |
|  | **RE-WU_ARDS_** | 166.13 | 30.32 |  |  |  |  |  |  |

Note: Data presented as mean ± standard deviation. *Significant difference at *p* < 0.05. PImax _I_ - maximal inspiratory muscle strength at rest, PImax _IV_ - maximal inspiratory muscle strength after the test, PEmax _I_ - maximal expiratory muscle strength at rest, PEmax _IV_ - maximal expiratory muscle strength after the test. WU_CON_ - warm-up in water, WU_ARDS_ - warm-up in water with ARDS, RE-WU_ARDS_ - warm-up in the water with application of ARDS on land during the transition phase between warm-up and swimming test.
