# Supplementary material for: Hypercapnic warm-up and re-warm-up–A novel experimental approach in swimming sprint
Source: PLoS One. 2025 Jan 29;20(1):e0314089. doi: 10.1371/journal.pone.0314089 (PMC11778800; doi:10.1371/journal.pone.0314089)
Supplement: Attachment — Submitted filename: Response to Reviewer 1_final.docx [file pone.0314089.s002.docx]

**Answer to Reviewer 1.**

Dear Reviewer,

thank you for your comments and suggestions. We appreciate your contribution for improving the quality of this manuscript. All changes have been made in "track the changes" mode in the main text. Below, we show detailed answers on your comments.

General comment:

The authors provided us an attempt to compare three warm-up routines and see if the effect of using a snorkel with added respiratory dead space would be beneficial for a 50 m maximal effort. This is an interesting approach with and a good idea to bring from the research area to the pool deck. Still, I make my reservations about what authors conclude by checking their data. To maintain the scientific coherency, I would suggest them to rethink what they want to conclude. Following you can find specific comments for improvement.

Specific comments:

Reviewer: The short title doesn’t match with your full title.

Answer: For a better matches, the main title has been edited to highlight hypercapnia caused by the use of the ARDS mask

Action: Change the main title: Hypercapnic warm-up and re-warm-up – a novel approach in swimming sprint Hypercapnic warm-up and re-warm-up – a novel approach in swimming sprint

Change the short title: Effects of hypercapnic warm-up in sprint swimming

Reviewer: Line 75-77: You say that one of the main goals to warm-up under hypoxia is to increase the number of red blood cells to increase oxygen transportation. But here you used a 50 m test that is mostly done under anaerobic conditions. Do with think that this is the best justification for your research gap?

Answer: We agree, it lead to misinterpretation in the introduction.

Action: This section was shortened with following final conclusion as gap for next paragraph “It should be mentioned that Robertson et al. [15] examined swimming performance during the 400m test, in which the aerobic energy system is dominant [17]. However, for improving anaerobic swimming performance (eg. in the 50m swimming sprint) other mechanisms should be stimulated during re-warm-up phase.”

Reviewer: Your participants section was not written based on inclusion and exclusion criteria. Can you rewrite considering this?

Answer: Of course, it should be mentioned in this section.

Action: This section was improved. We added sentence one complementary sentence ”They trained regularly eleven times per week“ and other about exclusion criteria: The exclusion criteria were as follows: 1) injuries three months before an experiment; 2) diagnosed asthma symptoms; and 3) smoking cigarettes. Additionally, we wrote some information about a priori sample size examining “The required sample size was determined by The G*Power software (version 3.1.9.2; Kiel University, Kiel, Germany).”

Reviewer: Please change “body weight” by “body mass” over the text since you express the value in kg.

Answer: Of course.

Action: Done

Reviewer: Line 118-120. Probably there is some formatting typo here.
Answer: You are right. It was changed.

Reviewer: I know what RBC, WBC and HGB is. But this should be described somewhere in the text.
Answer: We agree, it was changed.

Action: Added “RBC - erythrocytes; WBC - leukocytes; HGB – hemoglobin concentration;” under table.

Reviewer: Before using the snorkel with ARDS, you should have tested it in terms of kinematic and efficiency constraints to ensure the effort will be the same.

Answer: During previous studies after swimming with the ARDS apparatus, subjects were individually interviewed to ask if there was any discomfort in wearing a snorkel. The authors found that during low-intensity swimming used apparatus did not affect movement's kinematic and swimming efficiency (Szczepan et al. 2020; Szczepan et al. 2020; Szczepan et al. 2022).

Action: Improved.

Reviewer: Probably your RE-WUARSD needed to present some intensity on dry-land near to that performed during the WUCon. Don’t you agree?
Answer: No, we don’t agree with you, because the same PASSIVE break was used for all conditions. The only variable was the use of ARDS mask in the RE-WU_ARDS_ protocol, which significantly affected the change of acid-base balance, provoking the state of tolerated hypercapnia

Reviewer: Why use 20min of passive restitution and not less or more?
Answer: A 20-minute passive break based on the West et al. (2013) study was used (https://pubmed.ncbi.nlm.nih.gov/22789310/).

Reviewer: Line 169-170. Some confusing was made here. If the 50 m time trial was done without the ARDS, why mentioning the device at this stage? If they not used it in the maximal test you should clarify it.
Answer: We agree with this comment, writing in this format misleads the reader. This has been changed.

Reviewer: At any point of your results you show to us PO2 values. Why is that?
Answer: The data was supposed to be included in the supplement due to the lack of statistical differences, but for full reasoning we put the data in tables.

Action: Table 2 was enriched with pO2 analyses.

Reviewer: One of your findings is that “20 minutes of breathing through a 1200 ml added respiratory dead space volume mask during re-warm-up led to a reduction in the 50 m swim time”. I cannot agree with you, because form as statistically point of view no differences were found between the normal condition and RE-warm-up regarding the 50 m time (p = 0.9). The time differences are so tiny that can be attributed to the short effort or to the swimmers competitive level (high level swimmers are more able to maintain consistent results). At the end, if the use of ARDS is more effective in a re-warm-up phase comparing to a warm-up phase. But this is not observed when comparing to the normal condition (without the use of a ARDS). At any point in your results I found difference between re-warm-up and the normal condition (except for the PCO2 at phase III, still T50 was not different). So, coaches can retain from your findings that the use of a ARDS not changes so much the swimmers physiological capacity for a 50 m effort. Do you agree? Probably in longer efforts this will have another effects on race time. Do you agree?

Answer: We partially agree with you about overconfident inferences. However, when analyzing individual times, we observed a reduction in time in 6 out of 8 participants. Observing the reduction in swimming time by 0.3 seconds at a distance of 50 meters as a result of using one variable, which was the ARDS mask during the re-warm-up phase, it is difficult from a practical perspective as a trainer not to consider it as an effective method to improve swimming performance. Probably, the use of the RE-WUARDS phase before longer distances could cause greater effects, similar to Robertson's study, which proposed apnea phases before 400m. Future studies using ARDS are planned to be carried out at distances above 400m.

Action: We changed the part of Conclusion: Breathing through the added respiratory dead space volume mask of 1200 ml during the 20-minute passive sitting in the re-warm-up phase is an effective method of improving readiness for maximal effort in swimming in 50 m time trial front crawl, as opposed to breathing through ARDS during the active warm-up in the water. Although not statistically significant, the observed trend of reduced time trial among participants in the trials after RE-WU_ARDS_ compared to the control condition, the effect obtained during the warm-up phase can be successfully used in training conditions and sports competition of swimmers to improve performance.

Reviewer: For my interpretation you cannot say that there was no “muscle fatigue”. At any point of your intervention you used any EMG measures to ensure that assumption. So, you should have some care on that.

Answer: You are right, thank you for your suggestion.

Action: We added “Thuse, the tube used in the current study with a volume of 1200 ml and
a diameter of 2.5 cm did not seem to be a stimulus that can affect respiratory muscle fatigue. However, electromyography should be considered to confirm this in the further investigations.”

Reviewer: Line 472: “unpublished”. What do you mean? This is not a scientific way to present some findings.
Answer: That was a mistake. It has been corrected.

Sincerely,

Authors
