# Supplementary material for: Hypercapnic warm-up and re-warm-up–A novel experimental approach in swimming sprint
Source: PLoS One. 2025 Jan 29;20(1):e0314089. doi: 10.1371/journal.pone.0314089 (PMC11778800; doi:10.1371/journal.pone.0314089)
Supplement: Attachment — Submitted filename: Responses to Reviewer 2.docx [file pone.0314089.s003.docx]

**Answer to Reviewer #2:**

**Reviewer:** Dear Authors, thank you for considering my suggestions and incorporating them into the manuscript, which is globally improved, congratulations. Below are some specific suggestions with line indication.

**Answer:** Dear Reviewer, thank you very much for your time, all comments and contributions to improving our article.

**Action**: Your comments have greatly improved our article. We considered every point and hope our changes and response will prove to be satisfactory.

**Reviewer:** 148 &ndash; Please revise the format of the formula.

**Answer**: Of course.

**Action**: Done.

**Reviewer:** 149 &ndash; SD was previously abbreviated. Please revise these details in all manuscript.

**Answer**: Of course.

**Action**: Done.

**Reviewer:** 151 &ndash; &ldquo;p&rdquo; suggested in italic. Please revise these details in all manuscript (another example line 344).

**Answer:** Of course.

**Action**: Done.

**Reviewer:** 208, 209 &ndash; Please consider not placing the figures together in the manuscript, but immediately after their introduction in the text.
**Answer**: According to the journal's instructions, we must upload the graphics in a separate file.

**Action:** No action requirement

**Reviewer:** 229-230 &ndash; Please revise city and country.

**Answer**: Of course.

**Action:** Done.

**Reviewer:** 325 &ndash; Please revise the table 2 content and standardize the text alignment format (in this version text is centered but also at left). Please consider the same in tables 3, 4, and 5.
350-351 &ndash; Please revise. 395 &ndash; Please place space between value and unit.

**Answer:** We apologize for these oversights.

**Action**: This has been corrected.

**Reviewer:** 417-445 &ndash; Please consider splitting the paragraph to improve readability. Same in 448-472.

**Answer**: Thank you.

**Action:** This has been changed.

**Reviewer:** 416 &ndash; Without space / 447 with space / 482 different format. Please standardize.

511 &ndash; Abbreviations suggested. 586 &ndash; Please revise &ldquo;ARDSV&rdquo;.
**Answer**: Of course.

**Action:** It was changed.

**Reviewer:** 599-602 &ndash; Please revise the sentence considering a clear message. It is suggested to revise all the content of the conclusions section considering the results of the study, and providing the readers with clear and direct messages, if possible, with practical application.
**Answer**: Thank you for this suggestion. This fragment has been edited.

**Action:** Breathing through the added respiratory dead space volume mask of 1200 ml during the warm-up in the water or the 20-minute passive sitting in the re-warm-up phase did not significantly affect the reduction in time of the 50 m time trial front crawl. However, the use of ARDS during the re-warm -up phase is an effective method of improving readiness for maximal swimming effort in the 50 m time trial of front crawl, as compared to breathing through ARDS during the active warm-up in the water. Although not statistically significant, there was a trend of reduce time trial after RE-WUARDS. The effect obtained by using the ARDS during the re-warm-up phase can be applied in training and competitive sports conditions for swimmers to improve performance.

**Reviewer:** 637 &ndash; Please revise all the references format. Some examples: Ref 1 title in uppercase, ref 2 in lowercase; Ref 22 information is missing (journal, pages, and other); Same in ref 26.

**Answer:** Of course.

**Action:** Done.

Best regards,

Authors
