# Supplementary material for: Hypercapnic warm-up and re-warm-up–A novel experimental approach in swimming sprint
Source: PLoS One. 2025 Jan 29;20(1):e0314089. doi: 10.1371/journal.pone.0314089 (PMC11778800; doi:10.1371/journal.pone.0314089)
Supplement: Attachment — Submitted filename: Responses to Reviewer 3_round3.docx [file pone.0314089.s004.docx]

**Answer to Reviewer #3:**

**Reviewer:** Although conducted with rigor and concern for detailing all procedures, some questions are dubious or misinterpreted. My main consideration concerns the analysis of statistical results in which the p value associated with Fischer's exact test was not significant (time in the 50 m test, for example), but post-hoc indicated a difference. Now, if the ANOVA p value (p associated with Fischer's exact test) indicates no difference, post-hoc analyzes are not even sought. It is a basic principle of statistics. Therefore, it cannot be said that there was an improvement in performance in 50 m with the hypercapnia protocol.

**Answer:** Thank you for your insightful comments on our manuscript regarding the presentation of statistical measures in Table 2 and 3. We appreciate your attention to detail and your suggestions for improving the clarity and robustness of our data presentation. Upon reviewing your comments, we would like to provide some rationale for our choice to maintain the presentation of results. We removed the analysis of interactions between conditions from Table 2 and Table 3. If the analyses showed statistical significance in the main effect, we performed a detailed analysis of the interaction between the conditions and the results were described in the text (line 354-399). We believe that these measures hold a stronger relevance to the practical aspects of swimming and are more comprehensible to the target audience of this scientific communication. In conclusion, we appreciate your suggestion, on the basis of which we have corrected the presented results, so that, despite the lack of statistical significance of some parameters, we present a trend of changes useful to the practical needs and level of understanding of our target group in the swimming community. Every effort has been made to clarify in the text that the effects of using the ARDS mask in the various stages of the warm-up should be interpreted with caution. Thank you again for your insightful review and we remain open to any further suggestions and feedback.

**Action:** Extensive changes have been made to the results and disscussion sections.

**Reviewer:** Furthermore, what is the purpose of testing regressions if the coefficients are not clearly presented or discussed?

**Answer:** This study has also demonstrated several factors can potentially explain the time of performance The strongest predictor variables of time trial time t_25(2)_ in the regression model were La^-^, HCO_3_^-^, H^+^ (*p*<0.05), and the strength of the relationship between model and the dependent variable was at the 93% level.

**Action:** The regressions analysis descriptions has been added to the statistical analysis, results, and discussion section.

**Reviewer:** To perform the regressions, was the Durbin-Watson test applied?

**Answer:** The Durbin-Watson value has been added into the text.

**Action:** See sections: Statistical analyses/Results.

**Reviewer:** What variable input model? Have VIF coefficients been analyzed?

**Answer:** The information regarding to dependent (explained) variables and independent variables (explanatory) was applied into the text. The VIF value has been added as well.

**Action:** See sections: Statistical analyses/Results.

Best regards,

Authors
